# GM-CSF signalling blockade and chemotherapeutic agents act in concert to inhibit the function of myeloid-derived suppressor cells *in vitro*

**DOI:** 10.1038/cti.2016.80

**Published:** 2016-12-23

**Authors:** Tessa Gargett, Susan N Christo, Timothy R Hercus, Nazim Abbas, Nimit Singhal, Angel F Lopez, Michael P Brown

**Affiliations:** 1 Translational Oncology, Centre for Cancer Biology, SA Pathology and the University of South Australia, Adelaide, Australia; 2 Cytokine Receptor Laboratory, Centre for Cancer Biology, SA Pathology and the University of South Australia, Adelaide, Australia; 3 Cancer Clinical Trials Unit, Royal Adelaide Hospital, Adelaide, Australia; 4 Discipline of Medicine, University of Adelaide, Adelaide, Australia

## Abstract

Immune evasion is a recently defined hallmark of cancer, and immunotherapeutic approaches that stimulate an immune response to tumours are gaining recognition. However tumours may evade the immune response and resist immune-targeted treatment by promoting an immune-suppressive environment and stimulating the differentiation or recruitment of immunosuppressive cells. Myeloid-derived suppressor cells (MDSC) have been identified in a range of cancers and are often associated with tumour progression and poor patient outcomes. Pancreatic cancer in particular supports MDSC differentiation via the secretion of granulocyte-macrophage colony-stimulating factor (GM-CSF), and MDSC are believed to contribute to the profoundly immune-suppressive microenvironment present in pancreatic tumours. MDSC-targeted therapies that deplete or inhibit this cell population have been proposed as a way to shift the balance in favour of a tumour-clearing immune response. In this study, we have modelled MDSC differentiation and function *in vitro* and this has provided us with the opportunity to test a range of potential MDSC-targeted therapies to identify candidates for further investigation. Using *in vitro* modelling we show here that the combination of GM-CSF-signalling blockade and gemcitabine suppresses both the MDSC phenotype and the inhibition of T-cell function by MDSC.

Immune suppression has a critical role in the progression of tumours and in the resistance of tumours to treatments such as cytotoxic chemotherapy and immunotherapy. Tumours can inhibit tumour-associated antigen presentation, secrete immune-modulatory factors and recruit immune-suppressive cells.^1, 2^ Chronic inflammation in the tumour microenvironment results in the accumulation, activation and persistence of myeloid-derived suppressor cells (MDSC), which are in turn major contributors to immune suppression.^3^ These heterogeneous, immature, myeloid-derived cells have a range of phenotypes, including granulocytic and monocytic subtypes.^4^ The optimal panel of cell surface markers to define these cell populations is still under debate^5, 6^ but MDSC universally suppress the function and proliferation of effector T cells, which might otherwise be able to achieve targeted killing of tumour cells.^7, 8^ The number of MDSC circulating in the blood correlates with the clinical stage of some breast and gastrointestinal cancers, with increased percentages of MDSC associated with reduced overall survival.^9, 10^ Increased levels of MDSC have also been associated with a poor response to chemotherapy in humans.^11^

A striking example of the action of MDSC is observed in pancreatic cancer, which is characterised by profound immune suppression.^12^ Pancreatic ductal adenocarcinomas exhibit high numbers of MDSC but an absence of T cells, and it is believed that this immune suppression contributes to the aggressive nature of pancreatic cancer.^13^ In Australia in 2012, 2825 new cases of pancreatic cancer were diagnosed, and the disease has a 5-year survival of just 7% (http://pancreatic-cancer.canceraustralia.gov.au/statistics). The incidence of pancreatic cancer has slowly increased over the last 25 years; however, unlike the situation for other cancers, the mortality rate has not significantly improved. This highlights the need for further research into new treatments for pancreatic cancer.

It is increasingly clear that the MDSC population has a role in some of the most common and lethal cancers. Thus, it is important to identify targets within MDSC differentiation and functional pathways that offer potential targets for MDSC modulation. It has emerged that granulocyte-macrophage colony-stimulating factor (GM-CSF), a cytokine secreted by many tumours, is an important mediator of MDSC recruitment and differentiation. GM-CSF treatment alone is sufficient to induce an MDSC suppressive phenotype from human peripheral blood mononuclear cells (PBMC) *in vitro,*^14, 15^ and in mouse *in vivo* models.^16^ In patients, treatment with high doses of GM-CSF increased MDSC numbers.^17^ In mouse models of pancreatic cancer, it has been shown that locally applied anti-GM-CSF antibody, or knockdown of GM-CSF gene expression, prevents tumour growth following implantation and reduced MDSC infiltration of the tumour.^18, 19^ The inhibition of tumour growth following GM-CSF blockade depended on CD8^+^ T cells because depletion of these cells restored tumour growth. Thus, GM-CSF has a key role in the generation of MDSC and offers a potential target for therapy.

Recent reviews have identified the potential for MDSC-targeted therapy in cancer patients;^3, 20^ however, to date there is little clinical data. An effective MDSC-targeted therapy would not only be useful in potentially slowing tumour progression and improving the endogenous immune response to the tumour, but could also be used in combination with other novel immunotherapies, such as monoclonal antibody therapies, therapeutic cancer vaccines and the transfer of tumour-specific autologous T cells, which may likewise be inhibited by the presence of MDSC. One study has assessed the combined treatment of dendritic cell vaccination and All-trans retinoic acid, which promotes MDSC apoptosis, and found a reduction in MDSC numbers and an enhanced response to vaccination in cancer patients.^21^ However, there are also existing cancer treatments, as discussed below, that may interfere with MDSC differentiation, recruitment or function, and which have not yet been directly tested against MDSC in clinical trials.

The chemotherapeutic agents 5-Fluorouracil (5FU) and Gemcitabine (Gem) both reportedly exhibit effects on MDSC. In mice, and in *ex vivo* isolated human MDSC, both drugs induce apoptosis of MDSC,^22, 23^ but also activate pro-tumorigenic MDSC inflammatory pathways,^24^ which limits the efficacy of these drugs in mouse models. Conditioned media from Gem- or 5FU-treated pancreatic cell lines stimulates production of GM-CSF and other inflammatory factors such as interleukin (IL)-1β and cathepsin B, as well as promoting MDSC formation from monocytes.^24^ Patients given chemotherapy were also found to have lower frequencies of mature HLA-DR^+^ CD14^+^ cells and higher frequencies of granulocytic MDSC CD66b^+^ cells.^25^ This indicates that on their own, chemotherapeutic agents may not be sufficient to provide MDSC depletion. In line with this hypothesis, combinations of Gem or 5FU chemotherapy with cytokine killer cell therapy benefited pancreatic and renal cancer patients,^26^ and retrospective analysis identified that a decrease in circulating MDSC was associated with positive outcomes for these patients.

STAT3 is a key transcription factor in MDSC expansion and STAT3 inhibition of patient-derived MDSC-abrogated suppressor functions,^27^ suggesting that STAT3 inhibitors may also be useful as anti-MDSC therapeutics. For example, the tyrosine kinase inhibitor, Sunitinib (Sun), reduced MDSC numbers in patients reportedly via its inhibition of STAT3.^28, 29, 30^ Although Sun also inhibits T-cell proliferation and function *in vitro*,^31^ Sun-treated patients have improved expansion of tumour-infiltrating lymphocyte and intratumoural MDSC numbers are reduced, suggesting that MDSC normally suppress tumour-infiltrating lymphocyte expansion in the tumours of Sun-naive patients.^32^ These reports suggest that Sun and potentially other chemotherapeutic agents, such as Gem and 5FU, are candidates for use in combination with other immune-targeted therapies to inhibit the action of MDSC and to promote a favourable, tumour-rejecting immune response.

We have developed a GM-CSF mutant, E21R, that binds the alpha-chain of the GM-CSF receptor with normal low affinity but lacks agonist function because it is unable to bind the beta subunit of the GM-CSF receptor.^33, 34^ As a competitive antagonist, E21R neutralised the biological effects of GM-CSF *in vitro* and *in vivo*, resulting in blockade of GM-CSF-induced leukaemic cell proliferation and the apoptosis of leukaemic cells. Moreover, we translated our preclinical studies to a first-in-human, dose-escalation study in patients with advanced cancers of the colon, breast, lung and prostate. E21R was safe and associated with very mild side effects such that a maximum tolerated dose was not found. Effects on circulating leukocyte subsets were observed, and transient reductions in serum PSA levels were observed in two prostate cancer patients.^35^ We have also developed the GM-CSF-neutralising monoclonal antibodies 4D4 and 4A12 that block human GM-CSF activity *in vitro.*^36, 37^ Thus, E21R and the neutralising monoclonal antibodies 4D4 and 4A12 may be able to interfere with GM-CSF-dependent generation of MDSC. Given the role of GM-CSF in inflammatory diseases, GM-CSF -blocking therapies are currently in clinical trials in patients with rheumatoid arthritis and multiple sclerosis.^38, 39, 40^

The limited clinical data on MDSC-targeted therapy for cancer highlights an opportunity for investigating the anti-MDSC activity of routinely used anti-cancer drugs in combination with novel agents that target GM-CSF signalling. There are at least two biological processes *in vivo* where therapeutic interventions may be of use: (i) the differentiation of bone marrow progenitor cells into MDSC; and (ii) the intratumoral immunosuppressive functions of MDSC. The GM-CSF model of *in vitro* MDSC differentiation described by Lechner *et al.*^14^ and Bayne *et al.*^18^ provides a culture system to investigate: (i) GM-CSF-mediated induction of PBMC into MDSC; and (ii) the inhibitory effects of MDSC on autologous T-cell proliferation and cytokine secretion. In this study, we have specifically investigated whether blockade of GM-CSF signalling by a receptor antagonist or neutralising antibodies as single agents or in combination with commonly used anti-cancer drugs, will suppress either the induction of MDSC from PBMC, the T-cell inhibitory effects of MDSC, or both. The results of this study have identified novel combination therapy regimens that should be further investigated for their potential to re-programme the immune-suppressed, pro-tumour environment associated with MDSC-inducing tumours such as those found in patients with pancreatic cancer.

## Results

### Myeloid subpopulations in peripheral blood and in cells subjected to MDSC differentiation culture *in vitro*

**Figure 1:**
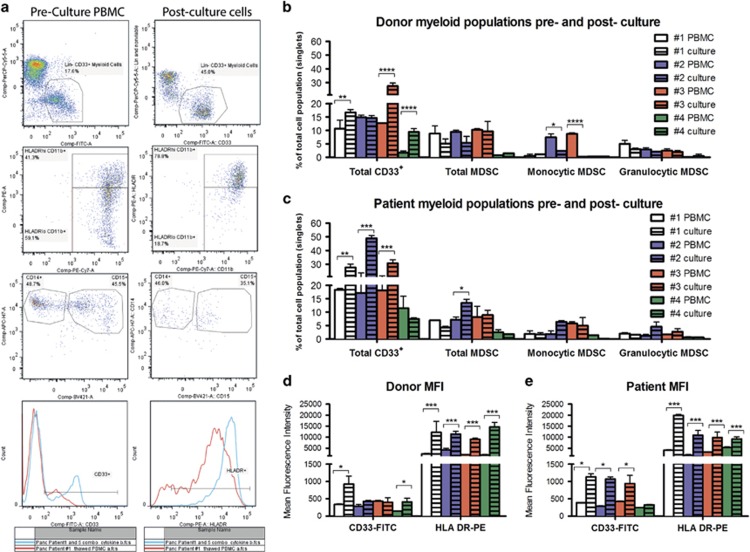
Identification of myeloid-derived cell populations by flow cytometry for four healthy donors and four pancreatic cancer patients. (**a**) Gating strategy to identify CD33^+^ myeloid subsets and representative CD33 and HLA-DR histograms for Patient #2 PBMC (Left hand panels) and Patient #2 PBMC cultured in combined IL-6 and GM-CSF cytokines (right hand panels). Myeloid cell population of (**b**) healthy donors and (**c**) pancreatic cancer patients in peripheral blood pre-culture (unpatterned columns) and 7 days post culture (striped columns) in the presence of 10 ng ml^−1^ each of GM-CSF and IL-6. Mean fluorescence intensity of CD33-FITC and HLA-DR-PE staining for CD33^+^ myeloid cells from (**d**) healthy donors, and (**e**) pancreatic cancer patients pre- and post culture. Data for patients 5–8 are shown in Supplementary Figure 1. Data were analysed by two-way ANOVA and Bonferroni multiple comparison post tests. Statistical significance is represented on graphs as *P⩽0.05, **P⩽0.01, ***P⩽0.001.

PBMC cryopreserved from healthy donors or untreated metastatic pancreatic cancer patients were thawed and cultured for 6–7 days in the presence of GM-CSF and IL-6 under conditions that have previously been defined as promoting MDSC differentiation.^14^ Freshly thawed PBMC (uncultured) were also analysed by flow cytometry for myeloid populations circulating in the peripheral blood. In this study, we used cells derived from both healthy donors and pancreatic cancer patients to allow for the possibility that patient-derived cells may have greater MDSC frequencies, or be less responsive to therapeutic intervention because of a more suppressive immune phenotype. We identified the following viable cell subsets by flow cytometry: (i) mature myeloid cells Lin^−^, CD33^+^, HLA-DR^+/hi^; (ii) immature myeloid cells (MDSC) Lin^−^, CD33^+^, HLA-DR^−/lo^, CD11b^+^; (iii) monocytic MDSC (moMDSC) Lin^−^, CD33^+^, HLA-DR^−/lo^, CD11b^+^, CD14^+^; and (iv) granulocytic MDSC (grMDSC) Lin^−^, CD33^+^, HLA-DR^−/lo^, CD11b^+^, CD15^+^, CD66b^+^ (Figure 1a).

In the MDSC differentiation cultures, we observed that more large adherent cells were present in most healthy donor and patient samples after cells were cultured with GM-CSF and IL-6, compared with proportions in peripheral blood, and the total proportions of CD33^+^ cells significantly increased in three of four donor cultures and six of eight patient cultures (Figures 1b and c and Supplementary Figure 1). Less-consistent changes were observed in the various MDSC subsets after culture in GM-CSF and IL-6, when compared with the frequencies in peripheral blood; the frequencies of the total MDSC subset increased significantly in three of eight patient cultures, whereas the moMDSC frequency decreased significantly in two of four donor cultures. The frequencies of the grMDSC myeloid subset were observed to be similar to the frequencies in peripheral blood, although two of eight patients had a greater proportion of grMDSC after culture in GM-CSF and IL-6. For the majority of donor and patient samples, the mean fluorescence intensity (MFI) of CD33 and HLA-DR also significantly increased following GM-CSF and IL-6 culture, indicating higher expression of these molecules on positive cells (Figures 1a, d and e and Supplementary Figure 1). Significant differences in the frequency of the various myeloid cell subsets between healthy donor and patient groups were not observed; however, it was noted patient samples did have a trend towards an elevated proportion of total CD33^+^ cells in their blood, and after culture in GM-CSF and IL-6, compared with donors. Thus, our data agree with previous reports that GM-CSF and IL-6 culture supported and promoted myeloid cell differentiation and the generation of the MDSC subset.

### Effects of GM-CSF-signalling blockade and chemotherapeutic agents on defined myeloid and MDSC populations

Next we investigated the effect of three GM-CSF blocking molecules: the neutralising monoclonal antibodies 4D4 and 4A12 and the GM-CSF antagonist E21R,^33, 34, 35^ as well as the chemotherapeutic agents, Gem, 5FU and Sun, on the myeloid cell phenotype of cells grown in MDSC differentiation cultures containing IL-6 and GM-CSF. After performing titrations of each agent in culture we found substantial toxicity of Sun above 1 μM, Gem and 5FU above 10 μM and E21R above 10 μg ml^−1^, and so excluded these concentrations from further analysis.

**Figure 2:**
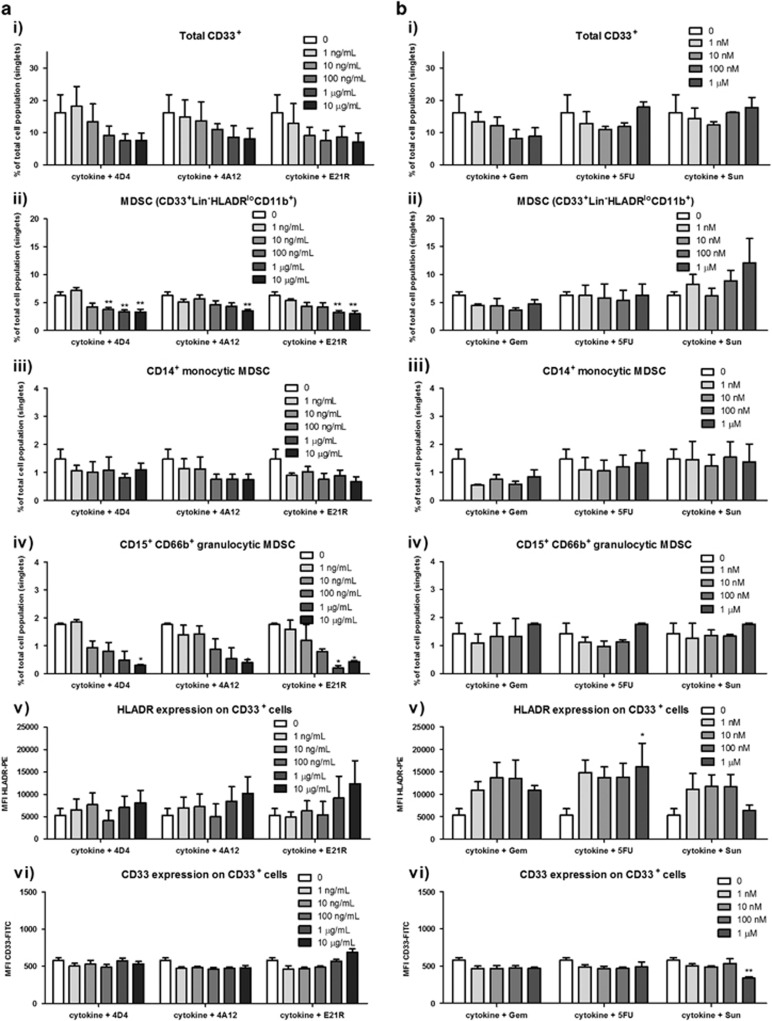
Determination of effective concentrations of GM-CSF-targeted therapeutics and chemotherapeutics on MDSC differentiation. PBMC from healthy donors and pancreatic cancer patients were cultured for 7 days at a concentration 5 × 10^5^ per ml in 10 ng ml^−1^ each of GM-CSF and IL-6 in the presence of increasing concentrations of (**a**) GM-CSF targeted therapeutics, 4D4 or 4A12 (anti-GM-CSF mAb), E21R (GM-CSF-antagonist) or (**b**) chemotherapeutics, Gem, 5FU and Sun. Flow cytometry was used to assess the frequency of five myeloid populations of interest (**i**) Total CD33^+^ population; (**ii**) Lin^−^ CD33^+^ HLA-DR^lo/−^ CD11b^+^ MDSC; (**iii**) CD14^+^ monocytic MDSC; (**iv**) CD15^+^ CD66b^+^ granuolcytic MDSC, and (**v**) the MFI for HLA-DR-PE within the CD33^+^ population and (**vi**) the MFI for CD33-FITC within the CD33^+^ population. Shown are pooled data for donors 1, 3 and 4. Data were analysed by two-way ANOVA and Bonferroni multiple comparison post tests. Statistical significance is represented on graphs as *P⩽0.05, **P⩽0.01.

Increasing concentrations of 4D4, 4A12 and E21R had no significant effect on the total CD33^+^ myeloid population, or on the CD33 MFI, suggesting that IL-6 may be sufficient to support this population in culture (Figure 2a). In keeping with this suggestion, cells cultured in IL-6 alone displayed myeloid cell subsets at similar frequencies to those differentiated in the presence of IL-6 and GM-CSF (not shown). GM-CSF blockade did however reduce MDSC frequency, and showed a trend towards decreasing the frequency of the grMDSC subset, which reached significance for E21R at the highest concentration. E21R treatment also resulted in a trend towards increasing HLA-DR expression on MDSC. Treatment with the chemotherapeutic agents on their own had no significant effect on the frequencies of total CD33^+^ myeloid cells or the MDSC subsets. 5FU treatment significantly increased HLA-DR expression and the other chemotherapeutic agents showed a similar trend, suggesting these agents may promote maturation of the myeloid subset.

**Figure 3:**
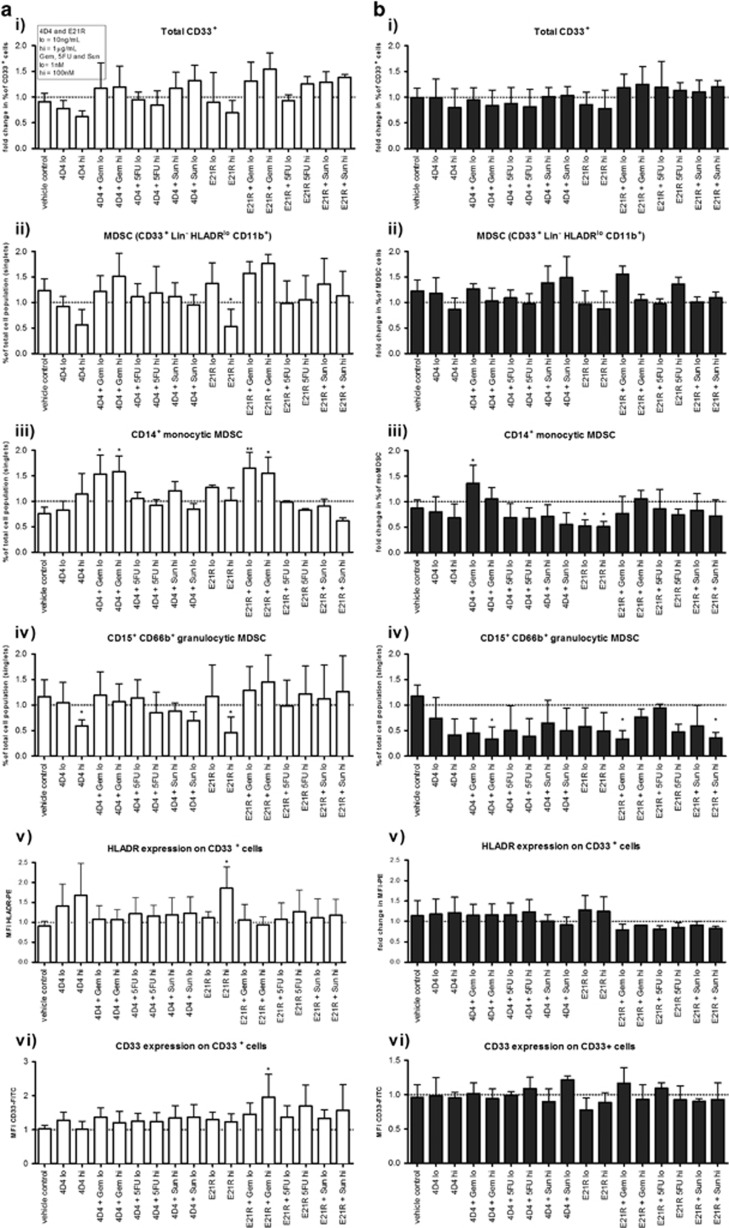
Combinatorial effects of GM-CSF-signalling blockade and chemotherapeutic agents on myeloid cell phenotype. MDSC differentiation cultures were performed as described in low (10 ng ml^−1^) or high (1 μg ml^−1^) concentrations of anti-GM-CSF antibody/antagonist and low (1 nM) or high (100 nM) concentrations of chemotherapeutic drugs. Graphs show the fold-change in phenotype compared with cells derived from differentiation cultures in the absence of signalling blockade or chemotherapeutic agents. (**a**) Healthy donor cultures (white bars) and (**b**) pancreatic cancer patient cultures (grey bars). Flow cytometry was used to assess the frequency of 5 myeloid populations of interest: (**i**) Total CD33^+^ population; (**ii**) Lin- CD33^+^ HLA-DR^lo/−^ CD11b^+^ MDSC; (**iii**) CD14^+^ monocytic MDSC; (**iv**) CD15^+^ CD66b^+^ granuolcytic MDSC; and (**v**) the MFI for HLA-DR-PE within the CD33^+^ population, and (**vi**) the MFI for CD33-FITC within the CD33^+^ population. Shown are pooled data for donors 1, 3 and 4 and patients 1, 2 3, 5, 6, 8. Data were analysed by two-way ANOVA and Bonferroni multiple comparison post tests. Statistical significance is represented on graphs as *P⩽0.05.

We also sought to determine whether the agents described above had a greater effect on the myeloid cell phenotype when used in combination. For these experiments we chose to examine the 4D4 antibody, the E21R antagonist as well as the three chemotherapeutic agents, and tested them in combination at a ‘low' (10 ng ml^−1^ or 1 nM) or ‘high' (1 μg ml^−1^ or 100 nM) concentration for each. The results are expressed as fold-change from the phenotype observed in GM-CSF and IL-6 cytokine cultures without therapeutic intervention (Figure 3). This revealed that, like single treatments, drug combinations had no impact on the frequency of the total CD33^+^ myeloid population. High concentrations of E21R alone significantly decreased the frequency of the MDSC population generated from healthy donors (as shown in the previous experiment Figures 2a and b ii), an effect that was lost when E21R was combined with chemotherapy (Figures 3a and b i–ii). The moMDSC frequency was significantly increased by the combination of 4D4+Gem (donors and patients) or E21R+Gem (donors only) (Figures 3a and b iii). The frequency of the grMDSC population decreased in response to 4D4 and E21R alone (donors) and 4D4+Gem, E21R+Gen and E21R+Sun combinations also significantly decreased the grMDSC population in patient samples (Figures 3a and b iv). There was a lesser effect of the drug combinations on CD33 or HLA-DR MFI, although E21R alone significantly increased the HLA-DR MFI and E21R+Gem significantly increased the CD33 MFI for donor samples (Figures 3a and b v–vi). Although the responses of cultured cells derived from donors and patients to the drug treatments varied, our results demonstrate that the drug combinations affected MDSC differentiation differently compared with their use as single agents. In general, the combination of GM-CSF targeted therapies with Gem appeared to promote the moMDSC phenotype and reduce the grMDSC phenotype, whereas the overall MDSC and CD33^+^ myeloid populations remained unchanged.

### Effects of GM-CSF-signalling blockade and chemotherapeutic agents on MDSC-mediated suppression of T-cell function

A remaining question in the MDSC field is how to appropriately define the MDSC population by surface marker expression.^1, 6, 41^ Although we have identified various myeloid subpopulations by flow cytometry and tracked the effect of drug combinations on these populations, one measure of whether these agents truly affect MDSC differentiation status is the ability of the resulting MDSC suppress effector T-cell function. To this end, we have sorted the entire CD33^+^ myeloid population after differentiation culture in the presence or absence of drug combinations and tested their ability to suppress T-cell proliferation or to secrete cytokines in response to stimulation.

**Figure 4:**
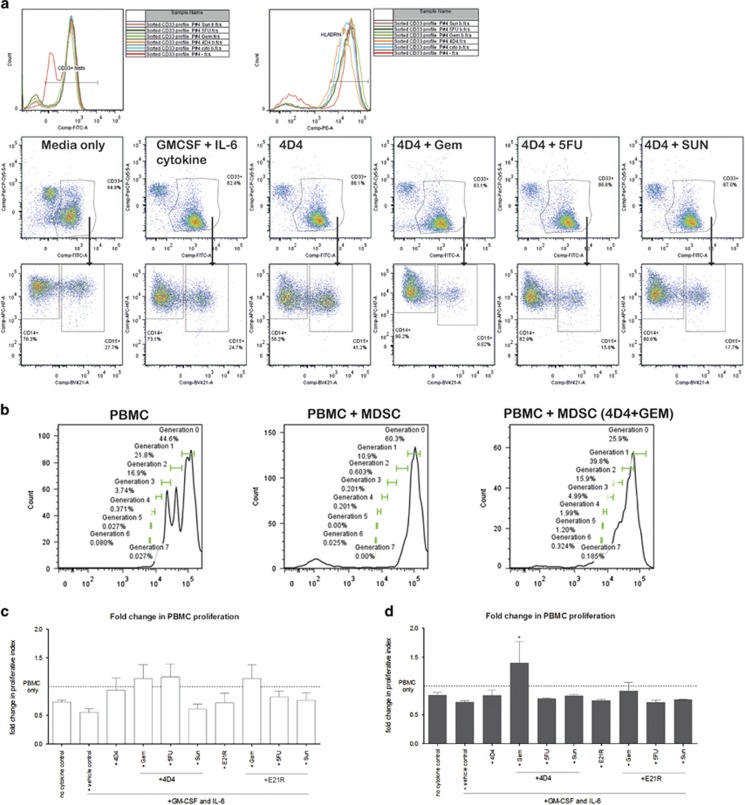

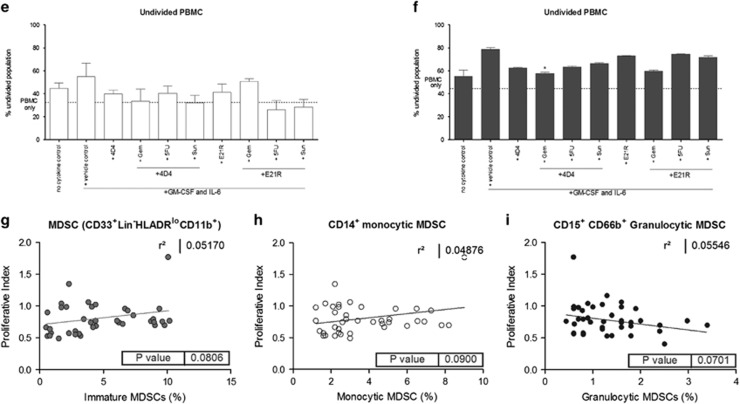
Effect of culture-derived MDSC on proliferation of autologous PBMC. Autologous Cell-Trace Violet-labelled PBMC and MDSC were co-cultured at a ratio of 2:1 for 5 days in the presence of 2 μg ml^−1^ anti-CD3 (plate bound) and CD28 (in solution). MDSC were isolated by CD33^+^ magnetic-activated cell sorting from cultures of PBMC in the presence of 10 ng ml^−1^ each of IL-6 and GM-CSF with or without 1 μg ml^−1^ 4D4, 1 μg ml^−1^ E21R, 100 nM Gem, 100 nM 5FU and 100 nM Sun. (**a**) Representative flow cytometry data of the phenotype of sorted CD33^+^ populations (CD33-FITC, Lin-PerCP-Cy5, CD14-APC-H7 and CD15-bv421 conjugated antibodies were used). (**b**) Representative PBMC proliferation plots (Cell-Trace Violet was detected in the ‘DAPI' 450/50 channel of the FACS Canto II). Fold-change in Proliferative Index of PBMC that have undergone division in presence of MDSC from (**c**) healthy donors (white bars) and (**d**) pancreatic cancer patients (grey bars). The mean PI of PBMC stimulated in the absence of MDSC is marked with the dotted line, proliferative index ranged from ~2 to 5 for individual donors. The percentage of PBMC that remained undivided after stimulation in presence of MDSC from (**e**) healthy donors (white bars) and (**f**) pancreatic patients (grey bars). The mean % undivided of PBMC stimulated in the absence of MDSC is marked with the dotted line. Shown are pooled data for Donors 1, 2, 3 and 4 and Patients 1, 2, 3, 5, 6 and 8. Data were analysed by one-way ANOVA and Bonferroni multiple comparison post tests. Statistical significance is represented on graphs as *P⩽0.05. Regression analysis of myeloid subset frequency and proliferative index: (**g**) immature myeloid subset Lin^−^ CD33^+^ CD11b^+^ HLA-DR^lo/−^ (MDSC); (**h**) monocytic MDSC subset Lin^−^ CD33^+^ HLA-DRl^o/−^ CD11b^+^ CD14^+^; (**i**) Lin^−^ CD33^+^ HLA-DR^−/lo^ CD11b^+^ CD15^+^ CD66b^+^.

Consistent with the results shown above, the sorted CD33^+^ myeloid populations from PBMC cultured in GM-CSF and IL-6, with or without cytotoxic drug treatment, had similar levels of CD33 expression and MDSC frequency but variable percentages of moMDSC and grMDSC (Figure 4a). Control cells grown in the absence of cytokine (that is, media only) had similar frequencies of myeloid subsets, but lower overall CD33 expression. In particular, we noted that cells grown in GM-CSF and IL-6 and the combination of 4D4+Gem or E21R+Gem had lower percentages of CD15^+^ grMDSC compared with other cultures.

Autologous Cell-Trace Violet-labelled PBMC were mixed with the sorted CD33^+^ myeloid cells at a ratio of 4:1 and stimulated with anti-CD3 and anti-CD28 for 5 days. Proliferation of CD3^+^ T cells was significantly inhibited by the presence of GM-CSF and IL-6 -cultured CD33^+^ cells from both donor and patient samples (Figures 4b–f), whereas CD33^+^ cells from the cultures without cytokine had less-suppressive activity, confirming that GM-CSF and IL-6 support an MDSC suppressive phenotype in culture.^14^ CD33^+^ cells sorted from cultures that included 4D4+Gem were less inhibitory to T cells when compared with CD33^+^ cells from cytokine cultures, as evidenced by an increased proliferative index (PI) and decreased proportions of undivided T cells. This result reached significance for the patient sample data, suggesting that the drug combination might reverse the MDSC suppressive phenotype. However, proliferation did not return to the levels observed for PBMC stimulated in the absence of CD33^+^ cells (Figure 4b). The same trend was observed for cells cultured in the presence of E21R+Gem. Regression analysis showed a weak positive correlation between moMDSC and PI, and a weak negative correlation between grMDSC and PI, however these associations were not statistically significant (Figure 4g).

We also assessed whether the inclusion of a single drug or drug combinations during the PBMC stimulation co-culture (in addition to inclusion during differentiation cultures) influenced CD33^+^ cell function and the suppression of T cells, but found no differences in the levels of suppression compared with those observed in PBMC co-cultures in the absence of therapeutic agents (not shown). These data suggest that the combination therapy has an effect on the initial differentiation of a myeloid suppressive subset rather than on the suppressive function of the myeloid cells in the presence of T cells.

**Figure 5:**
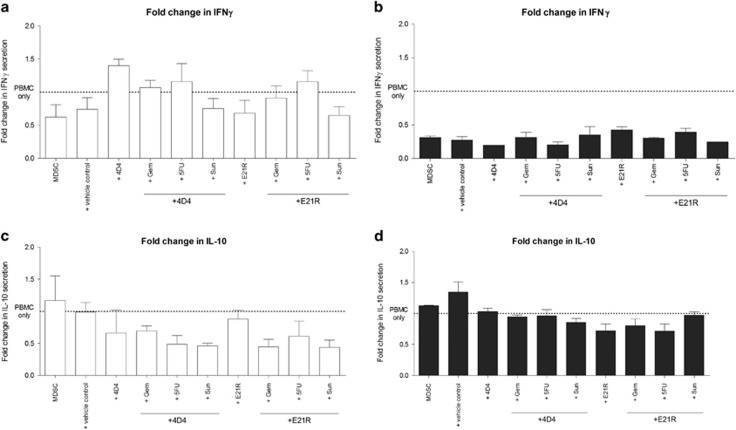
Effect of culture-derived MDSC on cytokine production by autologous PBMC. Co-cultures were established as described in Figure 4. Cell culture supernatants were collected at Day 5 and analysed by ELISA for IFNγ and IL-10. Fold-change in IFNγ secretion by PBMC that have undergone division in presence of MDSC from (**a**) healthy donors (white bars) and (**b**) pancreatic cancer patients (grey bars). The mean IFNγ secretion of PBMC stimulated in the absence of MDSC is marked with the dotted line. Fold-change in IL-10 secretion by PBMC that have undergone division in presence of MDSC from (**c**) healthy donors (white bars) and (**d**) pancreatic cancer patients (grey bars). The mean IL-10 secretion of PBMC stimulated in the absence of MDSC is marked with the dotted line. Shown are pooled data for Donors 1, 2, 3 and 4 and Patients 1, 2, 3, 5, 6, 8. Data were analysed by one-way ANOVA and Bonferroni multiple comparison post tests.

Supernatants were collected from PBMC and CD33^+^ co-cultures and analysed for cytokine levels (Figure 5). The mean interferon (IFN)γ levels secreted by stimulated PBMC in the absence of CD33^+^ cells are represented by the dotted line on each graph. As expected, co-culture of PBMC and CD33^+^ cells significantly reduced the levels of IFNγ detected in supernatant, suggesting MDSC also acted to inhibit T-cell IFNγ secretion following activation. Supernatants from co-cultures of healthy donor PBMC with CD33^+^ cells from cultures including single drug or drug combinations had a trend towards increased IFNγ, in particular for cells co-cultured with 4D4, 4D4+Gem, 4D4+5FU and E21R+5FU. However, none of these results reached statistical significance. Interestingly, supernatants from co-cultures of patient PBMC with CD33^+^ cells had significantly reduced IFNγ compared with co-cultures of healthy donor cells, and this was not altered when the CD33^+^ cells had previously been cultured with single drug or drug combinations. This result may indicate that the MDSC derived from patient samples have profound suppressive capacity, and are less responsive to therapeutic intervention than healthy donor-derived MDSC.

One proposed method of suppression by MDSC is mediated through IL-10 so we assayed supernatants to determine whether any changes in IL-10 levels were detected (Figures 5c and d). However, addition of CD33^+^ cells did not increase supernatant IL-10 levels over what was seen for PBMC in the absence of CD33^+^ cells (mean represented by the dotted line), and supernatants from co-cultures of PBMC and single drug or drug combinations cultured CD33^+^ cells likewise did not have significantly different IL-10 levels.

**Figure 6:**
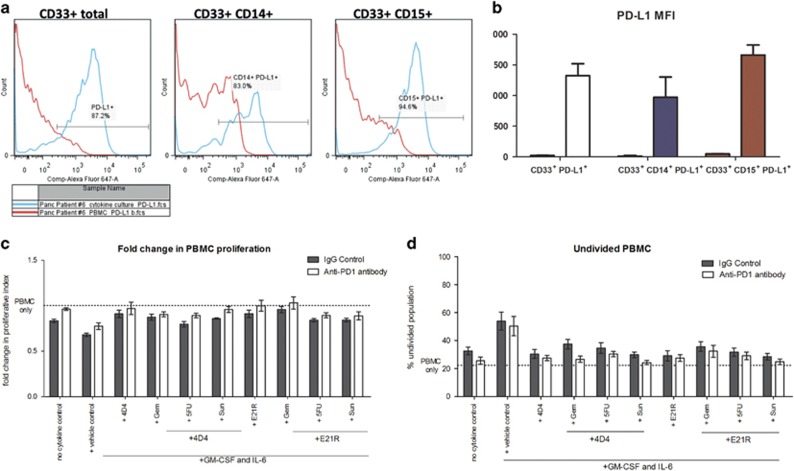
Investigation of PD-1 and PD-L1 interactions as a mechanism of MDSC-mediated suppression. Co-cultures were established and analysed as described in Figure 4. (**a**) Representative histogram of PD-L1 expression on circulating peripheral blood CD33^+^ cells and MDSC GM-CSF and IL-6 cytokine culture-derived CD33^+^ cells. (**b**) Mean fluorescence intensity of PD-L1-APC staining for myeloid cell subsets from healthy donors and pancreatic cancer patients pre- and post culture. (**c**) Effect of PD-1 blockade on the suppression of autologous PBMC proliferation by culture-derived MDSC as shown by fold-change in proliferative index or (**d**) the percentage of PBMC that remain undivided after stimulation. Shown are pooled data from three individual experiments (Patients 5, 6 and 8). Data were analysed by two-way ANOVA and Bonferroni multiple comparison post tests.

Another suggested mode of MDSC-mediated suppression is through interactions occurring between PD-1 on T cells and PD-L1 on MDSC.^42^ Given the recent interest in blockade of the PD-1/PD-L1 axis as a cancer therapy approach,^43^ we investigated PD-L1 expression on culture-generated MDSC, and tested whether PD-L1 had a functional role in suppressing T cells in the co-culture assay. We found that circulating MDSC in patient and donor blood had low to undetectable levels of PD-L1 expression, and that the GM-CSF and IL-6 differentiation culture significantly upregulated PD-L1 expression in CD33^+^ moMDSC and grMDSC subsets (Figures 6a and b). However, inclusion of anti-PD-1 blocking antibody in PBMC and CD33^+^ co-cultures did not significantly increase T-cell proliferation, although there was a trend towards an increased PI, and a decreased percentage of undivided T cells in the presence of anti-PD-1 antibody (Figures 6c and d).

## Discussion

In recent years, tumour evasion of the immune system via immune suppression has been identified as a hallmark of cancer.^1^ Immune suppression is mediated by complex factors including tumour-mediated cytokine secretion, inhibitory molecule expression and suppressive immune cell subsets.^5, 44^ MDSC are a heterogeneous cell subset that have been proposed to have an important role in immune suppression.^45^ In particular, MDSC have a key role in the resistance of pancreatic cancer to immune-mediated clearance. In models of pancreatic cancer, the tumour-secreted cytokine, GM-CSF, has been identified as promoting MDSC differentiation.^14, 18, 25^ For this reason, targeting MDSC is of interest as a potential immunotherapy for cancer,^46^ with the aim of reversing immune suppression and promoting an anti-tumour immune response. Accordingly, we have tested various GM-CSF-targeted and chemotherapeutic agents for their ability to interfere with MDSC differentiation or function *in vitro* so that we might identify candidates suitable for further preclinical and clinical testing in combination therapies.

We adopted a recommended antibody panel^6^ to track multiple myeloid subpopulations in culture and interrogate these subpopulations to determine which, if any, correlate with suppressive function. Our analysis included FSC and SSC characteristics to identify large cells, CD33^+^ HLA-DR^+^ mature myeloid cells, CD33^+^ HLA-DR^lo/−^ CD11b^+^ immature myeloid cells (referred to in this study as MDSC) as well as granulocytic-type (CD15^+^ CD66b^+^) grMDSC and monocytic-type (CD14^+^) moMDSC. We found that the addition of GM-CSF and IL-6 increased the frequency of total CD33^+^ myeloid cells, as well as the mean intensity of expression of CD33 and HLA-DR.

We investigated the effect of single GM-CSF targeted or chemotherapeutic agents, and combination treatments on the frequency of these various populations and concluded that the frequency of grMDSC was decreased by the combination of 4D4 or E21R with Gem, whereas the frequency of moMDSC was increased in response to the same combinations. Of note, in our hands CD15 was consistently co-expressed with intermediate levels of CD14 on cultured cells; we are not aware of previous reports of co-expression of these molecules within the CD33^+^ MDSC population. The combination of 4D4+Gem also affected the suppressive function of CD33^+^ myeloid cells when they were evaluated in co-cultures with autologous T cells, indicating that the grMDSC population may have more functional significance in this *in vitro* T-cell suppression assay. However, we were unable to attribute the suppression of T-cell function to any of the defined subsets using regression analysis. This suggests that function, rather than surface marker expression, remains the most accurate way to identify the MDSC population, and it is possible that the definitive MDSC surface molecule phenotype is yet to be fully identified. A limitation of this study is that we were not able to sort out defined moMDSC and grMDSC populations to include in the suppression assay and thus determine empirically whether one subpopulation in particular is responsible for the suppression of T-cell function. However, others have also reported that the granulocytic MDSC population in particular may be responsible for T-cell suppression. One clinical study found that grMDSC were negatively associated with circulating T-cell numbers and were associated with poor prognosis in HNSCC.^47^ Others found an association between grMDSC frequency and progressive disease or poor patient outcomes in patients with melanoma.^4^ Increased circulating grMDSC have also been identified in pancreatic cancer patients,^48^ whereas tumour-infiltrating myeloid cells are reported to be predominately grMDSC in patients with glioma.^49^

We have found that Gem in combination with GM-CSF blockade could reverse the suppressive phenotype of MDSC *in vitro*; however, the effect of Gem on this population in patients and preclinical models is still subject to debate, with some studies finding the drug increased MDSC numbers whereas others reported a decrease.^22, 23, 24^ A recent study has found that although Gem treatment of tumour cells increased the secretion of inflammatory factors and MDSC differentiation, inclusion of a GM-CSF-neutralising antibody could prevent myeloid cells from developing a T-cell suppressive phenotype.^25^ Our own study therefore supports this previous finding by confirming that Gem, in the presence of GM-CSF-neutralising antibodies or a GM-CSF antagonist, can reduce subsequent myeloid cell-mediated suppression of T cells. However, unlike previous publications,^32, 50^ we did not observe a reduction in MDSC function for myeloid cells treated with Sun, which may indicate this tyrosine kinase inhibitor mediates MDSC function by mechanisms that are not effectively modelled in this *in vitro* culture system. It is a limitation of this study that the *in vitro* assay cannot model the complex tumour microenvironment, which includes tumour cells and other resident immune cells that are also likely to have an impact on MDSC-T-cell interactions.

Although we found that the *in vitro* culture system adequately modelled induction of a suppressive MDSC-like population from PBMC, our experiments did not determine the mode of MDSC-mediated suppression observed in these assays. The two potential mechanisms that we investigated, IL-10 secretion and PD-L1/PD-1 interactions, did not significantly influence the levels of T-cell proliferation in the presence of MDSC, despite high levels of PD-L1 expression on culture-derived CD33^+^ cells. Others have a reported a mechanistic role for arginase activity and nitric oxide production in MDSC-mediated suppression,^8, 48, 51^ and this *in vitro* assay could offer a means to investigate how drug combinations can influence these potential suppression mechanisms. In addition, Bv8 and S100A8/9 are newly identified targets expressed by MDSC and MDSC-promoting cells and these molecules could also be evaluated as therapeutic targets using this assay.^52, 53, 54^

In this work we have compared donor-derived and patient-derived PBMC for their capacity to differentiate into MDSC, and then tested therapeutic combinations for the ability to interfere with MDSC function or phenotype. We did not observe significantly higher circulating MDSC in blood from pancreatic cancer patients compared with healthy donor blood. Nor did we find an increase in MDSC differentiation from patient-derived cells, and patient-derived MDSC were equally susceptible to therapeutic agents as measured by the phenotyping and proliferation assays. However, we did note that patient-derived MDSC produced a greater suppression of IFNγ secretion from autologous PBMC, suggesting patient-derived MDSC may have a more profound suppressive function compared with those that are derived from healthy donors. We also observed significant variability between individuals within the donor and patient groups, which hampered our statistical analysis. There is likely to be individual variation in the frequency and activity of MDSC as well as in their susceptibility to therapy.

The conclusions that can be drawn from this study are naturally limited by the use of samples from a small number of patients with a single tumour type, and a larger study may be able to confirm the myeloid subpopulation, which is responsible for T-cell suppression and which can be manipulated by combination therapies. It would also be useful to determine prognostic markers to predict responsiveness to MDSC-targeted therapy, for example the circulating CD15^+^ vs CD14^+^ MDSC subsets, or the neutrophil to lymphocyte ratio and this is again an area that requires further investigation in studies with larger patient numbers.

In conclusion, the *in vitro* model used in this study has allowed us to conduct an initial investigation of therapeutic targeting of MDSC. In agreement with another recently reported study,^25^ we found that GM-CSF blockade *via* a neutralising antibody or an antagonist in combination with the cytotoxic drug, Gem, can reverse MDSC differentiation and its suppression of T-cell function, therefore suggesting a candidate combination therapy worthy of further investigation. Preliminary clinical data demonstrate the safety and efficacy of GM-CSF cytokine and receptor-blocking antibodies in inflammatory conditions.^38, 39, 40, 55, 56^ Hence, together with our *in vitro* data, a clinical rationale exists for combining GM-CSF blockade with standard Gem therapy in pancreatic cancer patients, with the aim of limiting the MDSC differentiation occurring in response to chemotherapy-induced inflammatory mechanisms. Consequently, using this approach to overcome the immunosuppressive tumour microenvironment could increase the therapeutic effectiveness of immune inhibitors or adoptively transferred T cells.

## Methods

### Human PBMC

Peripheral blood samples of 40–80 ml were obtained from four healthy volunteers and eight metastatic pancreatic cancer patients with progressive untreated disease. Approval for this project was obtained from the RAH Human Research Ethics Committee (Approval #131208), and informed written consent was obtained from all study participants. Human PBMCs were purified by density gradient separation with Lymphoprep (EliTech Group, Braeside, VIC, Australia) and immediately used for flow cytometry to determine MDSC phenotype and to establish MDSC differentiation cultures, as well as cryopreserved for use in functional assays. Cancer patients #1–8 had Neutrophil:Leukocyte ratios of 5.1, 5.39, 27, 2.98, 3.24, 27.1, 6.2 and 2.2, respectively, Patients #4 and #7 were not included in pooled analyses because of poor yields of CD33^+^ cells in differentiation assays, a pre-established criteria.

### Antibody and chemotherapeutic treatments

The antibodies and GM-CSF antagonist were developed in house. The anti-PD-1 blocking antibody, pembrolizumab, was obtained from the residuum contained in infusion bags routinely administered to patients, and before discard. E21R is a mutant form of GM-CSF that binds to the alpha-chain of the GM-CSF receptor with low affinity and acts a competitive antagonist to neutralise GM-CSF signalling. 4D4 and 4A12 are anti-human GM-CSF-neutralising antibodies. An immunoglobulin G1 isotype control (1B5) was used as a negative control. The chemotherapeutic agents Gem, 5FU, Sun were purchased from Selleck Chemicals (Jomar Life Research, Scoresby, VIC, Australia) and stored as stock concentrations of 1 mM in dimethyl sulfoxide and further diluted in RPMI (Roswell Park Memorial Institute medium) before use. Equivalent concentrations of dimethyl sulfoxide in RPMI were used as vehicle controls. In combination experiments, the vehicle control was 100 ng ml^−1^ isotype control antibody and 0.1% (v/v) dimethyl sulfoxide. Treatments were used at the concentrations indicated in the figure legends.

### *In vitro* generation of human MDSC

Thawed PBMCs were cultured in advanced RPMI (Sigma Aldrich, Sydney, NSW, Australia) supplemented with 10% fetal bovine serum and 10 ng ml^−1^ of human GM-CSF (Life Technologies) and 10 ng ml^−1^ of human IL-6 (Life Technologies, Mulgrave, VIC, Australia). GM-CSF and IL-6 were stored as stock concentrations at 100 μg ml^−1^, and we noted that MDSC differentiation was highly dependent on the quality and activity of cytokine preparations—working solutions of 10 μg ml^−1^ and below were therefore freshly prepared directly before addition to the culture medium. PBMC were cultured in the presence of various anti-GM-CSF agents (E21R, 4D4, 4A12) in combination with chemotherapy (Gem, 5FU, Sun) as described in figure legends. PMBCs (5 × 10^5^ per ml cells per T25 flask or 2.5 × 10^5^ cells per well in a 48-well plate) and were fed every 4 days by replacing 50% of the media with fresh complete RPMI containing GM-CSF and IL-6 and the specified combination of agents.

### Isolation of MDSC via magnetic sorting and positive selection

On day 6–7 of PBMC culture, suspension and adherent cells were removed from T25 flasks (Detachin, San Diego, CA, USA; Genlantis and gentle cell scraping), collected by centrifugation and incubated with anti-human CD33 microbeads (Miltenyi Biotec, Sydney, NSW, Australia) for 15 min at 4 °C before being washed and resuspended in 500 μl magnetic-activated cell sorting buffer before application to magnetic-activated cell sorting LS Columns (Miltenyi Biotec). Cells were washed three times in magnetic-activated cell sorting buffer. To collect the attached CD33^+^ cells, the columns were removed from the magnet and washed twice with 5 ml magnetic-activated cell sorting buffer; an initial collection via gravity flow and a final wash expelled using the plunger.

### MDSC phenotyping

Thawed uncultured PBMC, the PBMCs cultured for 6–7 days, or the positively selected CD33^+^-purified cells, were assessed for the following anti-human surface antigens (BD Bioscience, North Ryde, NSW, Australia): CD33-FITC, CD11b-PeCy7, HLA-DR-PE, lineage markers (Lin; CD3, CD19, CD56 PerCP-Cy5.5), CD14-APC-H7, CD15-bv421 and CD66b-APC or PD-L1-AF647. MDSC surface marker expression was classified into three groups: monocytic MDSC (moMDSC) Lin^−^ CD33^+^ CD11b^+^ CD14^+^ HLA-DR^−/low^; granulocytic MDSC (grMDSC) Lin^−^ HLA-DR^−/low^ CD33^+^ CD11b^+^ CD15^+^ CD66b^+^; or immature MDSC Lin^−^ CD33^+^ CD11b^+^ HLA-DR^−/low^. Mature myeloid cells were defined as Lin^−^ CD33^+^ CD11b^+^ HLA-DR^+/hi^.

### T-cell proliferation

The suppressive function of MDSC was assessed by measuring proliferation of autologous T cells *in vitro*. Autologous T cells were thawed from cryopreserved PBMC stocks, and viability was assessed to ensure ⩾80% viability. Whole PBMCs were stained with 5 μM Cell-Trace Violet (Life Technologies) as per manufacturer's instructions, then incubated with 1 μg ml^−1^ purified anti-human CD28 (Miltenyi Biotec) for 15–20 min at 37 °C to allow binding. Whole PBMCs were seeded at 1 × 10^5^ cells per well into round-bottom 96-well plates prepared with adherent anti-human CD3 and soluble anti-human CD28 (purified, 2 μg ml^−1^; Miltenyi Biotec). The CD33^+^-purified cells from the various culture conditions were then added to the wells in different numbers to generate wells of 4:1 (PBMC: CD33^+^ cells) ratios, maintaining a consistent number of PBMCs among the wells. Some wells also received GM-CSF blockade or chemotherapeutics at the concentrations described in the text, or pembrolizumab at 10 μg ml^−1^. The plate was incubated for 5 days at 37 °C before supernatant and the cells washed and stained with anti-human CD3-PE and CD33-FITC (BD Bioscience) antibodies for flow cytometric analysis. Samples were run on a FACSCantoII flow cytometer (BD Bioscience) and populations were analysed using FlowJo version 7.6.5 (TreeStar Inc, Ashland, OR, USA) for PI and % undivided cells. Statistical analyses were performed as described in the figure legends.

### Measuring IFNγ and IL-10 secretion

Supernatants were collected at the end of assays measuring T-cell proliferation, and stored at −20 °C until use. Concentrations of cytokine were measured using the Human IFNγ and Human IL-10 ELISA kits (ELISAkit.com, Scoresby, VIC, Australia) as per manufacturer's instructions. Absorbance was read on a FLUOStar Omega microplate reader (BMG Labtech, Mornington, VIC, Australia) and values were used to calculate absolute concentrations before plotting the data.

### Statistical analysis

Data were analyzed using GraphPad Prism Version 6.0d. Data were analyzed by one-way analysis of variance (for single variable data) or two-way analysis of variance (for two variable data) and Bonferroni multiple comparison post tests. Significance is represented on graphs as *⩽0.05, *⩽0.01 and ***⩽0.001.
