# Supplementary figures and images for: GM-CSF signalling blockade and chemotherapeutic agents act in concert to inhibit the function of myeloid-derived suppressor cells in vitro
Source: Clin Transl Immunology. 2016 Dec 23;5(12):e119–. doi: 10.1038/cti.2016.80 (PMC5192067; doi:10.1038/cti.2016.80)

## Patient myeloid populations pre- and post- culture

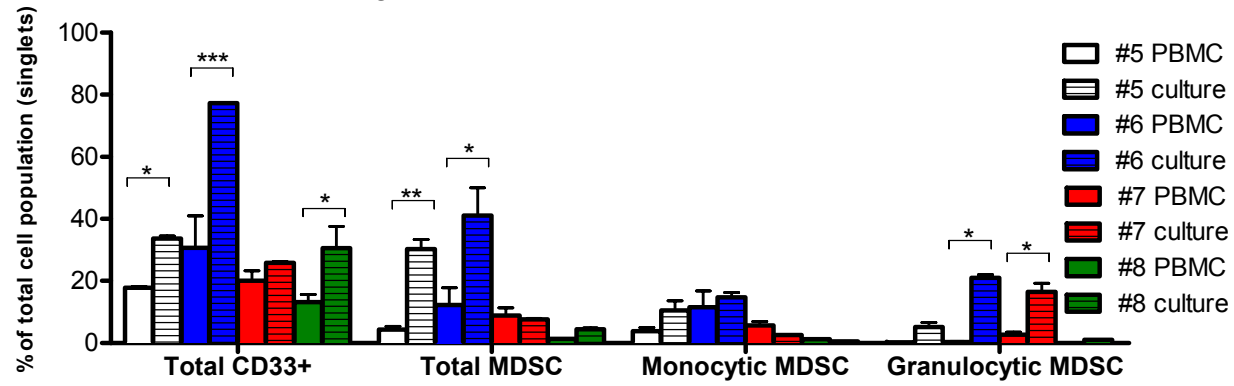

## Patient MFI

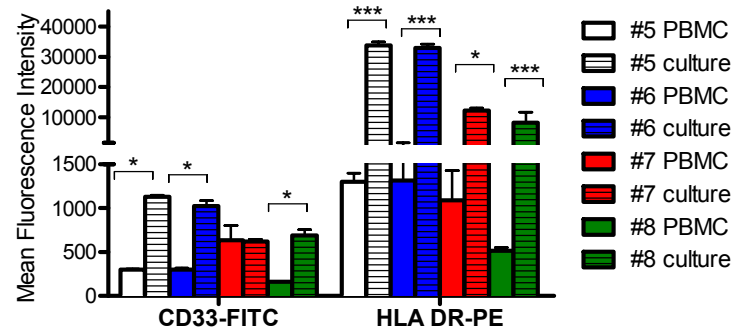

Supplement: Supplementary Figure [file cti201680x1.pdf]
